# Lung cancer screening: a mini review of the major trials and guidelines

**DOI:** 10.1590/1806-9282.2024S111

**Published:** 2024-06-07

**Authors:** Wolfgang William Schmidt Aguiar, Daniel Oliveira Bonomi, Francisco Martins, Clara de Andrade Pontual Peres, Arthur dos Santos Sena

**Affiliations:** 1 Brazilian Society of Thoracic Surgery – São Paulo (SP), Brazil.; 2 University of Pernambuco – Recife (PE), Brazil.

## INTRODUCTION

Lung cancer remains a notable global health concern due to its high incidence and mortality rates. In 2020, there were an estimated 1.8 million lung cancer-related deaths and 2.2 million new lung cancer cases, making it the leading cause of cancer death (18% of all cancer deaths) and the second most frequently diagnosed cancer in the world (11.4% of all cancer diagnoses)^1^.

Lung cancer often goes undetected until its advanced stages, with these late diagnoses contributing immensely to a poor prognosis. In most countries, the 5-year survival rate in patients with lung cancer is only 10–20%^1^. The presence of metastasis upon first diagnosis, indicating advanced disease, is the main cause of treatment failure, while patients diagnosed at an earlier stage, like stage IA, and adequately treated have significantly higher 5-year survival rates, exceeding 70%^2^. This underscores the importance of early diagnosis and appropriate treatment for better outcomes in lung cancer patients.

Exposure to risk factors is intimately linked to lung cancer etiology. The most important and prevalent risk factor is tobacco smoking, which accounts for 80–90% of lung cancer diagnoses, despite the fact that only about 15% of smokers develop this neoplasm. Tobacco smoke contains many carcinogens, causing the relative risk of lung cancer in a smoker to be around 20 times higher than the risk in a nonsmoker^3^. The global pattern of lung cancer incidence is related to the tobacco epidemic, and since the disease has poor survival and high fatality rates, its mortality is also associated with such an epidemic^1^. It is important to note that there are also other risk factors that can be associated with lung cancer, such as secondhand smoke, electronic cigarettes, pre-existing lung disease, occupational exposures, and oncogenic viruses^3^.

In this context, it is evident the importance of primary prevention of lung cancer, which consists of reducing smoking initiation, particularly in the younger population, and increasing smoking cessation, to achieve a reduction in risk and mortality^1,3^. It is also important to implement secondary prevention in people who are at high risk (current and former heavy smokers) to detect lung cancer in its earliest stages, when treatment, mainly surgical, is most successful^3^.

In this sense, significant effort was made to enhance early diagnosis and treatment for lung cancer in order to improve patient outcomes. Initially, in the 1970s, trials using chest radiography and sputum cytology to detect early lung cancer were performed, which proved to be ineffective in reducing its mortality. Later, in the 1990s, low-dose spiral chest computed tomography (LDCT) was shown to have potential usefulness in lung cancer screening (LCS)^2^. Since then, multiple international observational studies and randomized trials have been executed, confirming the efficacy of annual LDCT in reducing lung cancer mortality and thus serving as the basis for current guidelines concerning lung cancer prevention and screening^1,2^. In the present study, we aim to do a mini-review of the major trials and guidelines concerning lung cancer screening (LCS).

## METHODS

PubMed/MEDLINE and the Cochrane Library were searched for English-language articles published until August 2023, with the following descriptors: lung cancer; screening; diagnosis; smoking cessation; treatment. Our team also reviewed reference lists of pertinent articles and studies suggested by the review writers.

The aim was to find the most pertinent randomized controlled trials regarding screening for lung cancer with LDCT and guidelines about the same topic, published by different respected entities with a broad spectrum of different countries.

Two reviewers selected the trials and/or guidelines, taking into consideration the relevance, methodology, impact in the scientific community, quality of the journals, and range and respect of the entities when it came to the guidelines.

## RESULTS AND DISCUSSION

The first double-blind randomized controlled trial regarding LCS with statistically relevant results was the National Lung Screening Trial (NLST), which was also the largest trial ever performed in that matter, as shown in Table 1. It opened the door for discussion and research on early diagnosis and screening for lung cancer, considering that most of the research regarding that disease targets treatment options.

**Table 1:** Major trials about lung cancer screening and their results.

Trial	Study design	Number of participants	Target group (age and smoking status)	Summary of findings	Additional points
NLST (National Lung Screening Trial)	Participants randomly assigned to one of two screening groups: one group underwent LDCT annually for 3 years, and the other group underwent chest X-ray annually for the same period.	>53,000	55–74 years old, with a history of smoking for at least 30 years or had quit smoking within the past 15 years.	The results of the study showed that LDCT reduced lung cancer mortality by 20% compared with chest X-ray.	X
NELSON ((NEderlands Leuvens Screening ONderzoek)	Participants were randomly assigned to either LDCT group or the control group. The LDCT group received screening with low-dose computed tomography scans at baseline and after 1, 2, and 4 years.	>15,000	40–74 years old, who were current or former smokers with a smoking history of at least 10 cigarettes per day for at least 30 years or 15 cigarettes per day for at least 25 years.	The primary endpoint of the study was lung cancer mortality. The LDCT group had a significantly lower cancer mortality (up to 20%) rate compared with the control group.	X
UKLS (UK Lung Screen Trial)	Participants were randomly assigned to LDCT screening (periodicity defined according to the Wald Single Screen Design) or no screening (usual care).	4,055	50–75 years old with the risk score Liverpool Lung Project (LLPv2)≥4.5%	While the UKLS showed benefits in early detection, the study was not sufficiently large or long term to determine a direct impact on lung cancer mortality reduction.	Screening with LDCT resulted in a high proportion of lung cancers being detected at early stages. In the screened group, 87.8% of diagnosed cancers were at stage I or II. The trial shows, however, a proportion of false-positive results of 18.5% (nodules that were initially suspicious but later confirmed as benign).
LUSI (Lung Screening Intervention Trial)	Participants were recruited from the general population and randomly assigned to LDCT screening or no screening during 5 years.	4,052	50–69 years old, with eligibility criteria being defined by at least 25 years smoking of at least 15 cigarettes per day or at least 30 years smoking of at least 10 cigarettes per day, including ex-smokers who had stopped smoking not more than 10 years before invitation to screening.	Modeling by sex showed a statistically significant reduction in lung cancer mortality among women (HR=0.31 [95%CI 0.10–0.96], p=0.04), but not among men (HR=0.94 [95%CI 0.54–1.61], p=0.81) screened by LDCT.	X
MILD (Multicentric Italian Lung Detection)	Participants were randomized to annual or biennial LDCT, with a median screening period of 6.2 years or no screening (usual care).	4,099	49–75 years old, current or formers smokers (<10 years of quitting) of ≥20 packs/year without history of cancer in ≤5 years.	LDCT screening was associated with a significant 39% reduction in lung cancer mortality at 10 years (HR 0.61; 95%CI 0.39–0.95; p=0.017), as well as a nonsignificant 20% decrease in all-cause mortality.	The biennial LDCT arm showed a similar overall mortality (HR 0.80, 95%CI 0.57–1.12) and LC specific mortality at 10 years (HR 1.10, 95%CI 0.59–2.05), as compared with annual LDCT arm.

Those trials have all come to similar findings, showing that LDCT is a great choice for LCS, and it has the capability of reducing up to 20%, in some trials even more, of lung cancer-related mortality. That comes up as extremely enthusiastic for the scientific community that had, and still has, witnessed the dramatic cases of advanced lung cancer.

The trials showed, however, some points that need to be analyzed carefully before implementing a screening program, such as the presence of false-positives, which lead to unnecessary surgical intervention and patient-family anxiety, and the detection of lesions that may never become cancer, leading to overdiagnosis and overtreatment. Smaller trials in low- to middle-income countries have shown that the rate of false-positives increases significantly in tuberculosis-endemic areas. Those outcomes were minimized, though, with the performance of the screening in specialized centers with highly defined protocols, the analysis of an experienced multidisciplinary team, and the presence of a thoracic radiologist.

Alongside that, much has been speculated about the cost-effectiveness of LCS with LDCT, considering the cost of that screening for large populations. A systematic review from the Lung Cancer Journal, published in 2022, evaluates that matter. The review looked at 45 studies, including trials and modeling studies. 86.7% of the studies found screening with LDCT to be cost-effective, being optimal between the ages of 55 and 75 years, with a history of at least 20 packs per year.

Another aspect shown in the trials was that, in patients who were current smokers during screening, the smoking cessation rate was extremely higher compared to those that didn't undergo screening.

Considering all that, important societies and entities started publishing guidelines based on those trials; they can be seen in Table 2. Most of the guidelines have similar recommendations, with annual LDCT screening for risk groups as the standard. Also, specialized centers are recommended, as described.

**Table 2:** Guidelines for screening for lung cancer.

Guideline	Recommendations
USPSTF (United States Prevention Taskforce), 2021	Screening with annual LDCT in individuals between the ages of 50 and 80 years with a history of smoking at least 20 packs/year. The screening must be done in specialized centers with highly defined protocols, to minimize the rate of false positives and overdiagnosis.
European Society of Radiology+European Respiratory Society, 2020	Screening with LDCT in individuals aged 50–75 years with a smoking history of at least 20 packs/year and a quit time of less than 10 years. Should be done yearly for at least 3 years. The results must be interpreted by radiologists with expertise in thoracic imaging.
Brazilian Society of Pneumology and Phthisiology+Brazilian Society of Thoracic Surgery+Brazilian College of Radiology and Imaging Diagnosis, 2023	LDCT annually in individuals between 50 and 80 years old, who are current smokers or quit smoking in the last 15 years, with a smoking history of at least 20 packs/year.
Canadian Task Force on Preventive Health Care, 2016	Screening with LDCT in individuals aged 55–74 years with at least a 30 packs/year smoking history, who currently smoke or quit less than 15 years ago. Annual screening with LDCT up to three consecutive years. Screening should only be carried out in health care settings with expertise in early diagnosis and treatment of lung cancer.
National Comprehensive Cancer Network, 2022	LCDT screening in individuals aged between 55 and 77 years, with a >30 packs/year smoking history, who are current smokers or quit in the past 15 years. Or individuals with more than 50 years old, with a smoking history of > 20 packs/year, with additional risk factors.
Royal Australian and New Zealand College of Radiologists (RANZCR), 2021	Age between 50 and 74 years; 20 or more packs/year history of smoking tobacco; and, if former smoker, have quit within 20 years should undergo helical LDCT. To be involved in the program, participants should also be willing to receive counseling and participate in shared decision-making before screening.

Those guidelines evaluated important aspects of LCS, such as the difference in all-cause mortality, lung cancer mortality, and quality of life; effectiveness in different subgroups; effectiveness associated with frequency of screening; accuracy of screening with LDCT; harms associated with that; and other practices that should be encouraged to diminish the incidence of lung cancer, being able to minimize, in the future, the number of people in the risk groups.

One of those practices, encouraged by most of the guidelines, takes place in smoking cessation programs that should have a broad range for all the population, with multidisciplinary teams involving mental health care professionals and multiple strategies for smokers to quit smoking, as well as educational programs for nonsmokers. That increases tremendously the cost-effectiveness of screening, considering that the risk groups would become smaller and smaller with time.

## CONCLUSION

Even though the benefits of LDCT in LCS have been proven, the implementation of such programs still faces important challenges. The first one concerns continuing medical education programs, so all of the medical society becomes aware of the need for LCS, as it already happens in other neoplasms, such as breast cancer and colorectal cancer.

Another concern regards the stigma still present in the face of a lung cancer diagnosis, considering the intimate relationship with smoking and the consequent guilt and stress that the diagnosis may trigger in the patient and their family.

Also, understanding and developing culture-sensitive screening approaches is essential, especially when it comes to low- and middle-income countries, where infrastructure and access to healthcare may be a problem, and that must be faced with strategies such as public-private partnerships and the employment of mobile CT scanners.

At last, the results shown here must be seen with extreme hope that, in the future, hopefully in the short term, our community will be able to see fewer advanced lung cancer cases, with more early diagnosis, and an exponential reduction in smoking levels.
